# Collagen turnover biomarkers to predict outcome of patients with biliary cancer

**DOI:** 10.1186/s12876-025-03645-0

**Published:** 2025-02-04

**Authors:** Leonard Kaps, Muhammed A. Genc, Markus Moehler, Stephan Grabbe, Jörn M. Schattenberg, Detlef Schuppan, Rasmus Sund Pedersen, Morten A. Karsdal, Philipp Mildenberger, Annett Maderer, Nicholas Willumsen

**Affiliations:** 1 Department of Dermatology, University Medical Center of the Johannes Gutenberg-University, Mainz, 55128 Germany; 2 Department of Medicine II, Saarland University Medical Center, Saarland University, Homburg, 66421 Germany; 3 First Department of Medicine, University Medical Center of the Johannes-Gutenberg University, Mainz, Germany; 4 University Medical Center, Institute of Translational Immunology and Research Center for Immunotherapy, Johannes Gutenberg-University, Mainz, Germany; 5 Division of Gastroenterology, Beth Israel Deaconess Medican Center, Harvard Medical School, Boston, Mam USA; 6 Nordic Bioscience A/S, 2730 Herlev, Denmark; 7 Institute of Medical Biostatistics, Epidemiology and Informatics, University Medical Center of the Johannes Gutenberg University, Mainz, Germany

**Keywords:** Tumor marker, Extracellular matrix, Gastrointestinal cancer

## Abstract

**Background:**

The collagen-rich tumor stroma plays a crucial role in biliary tract cancer (BTC). Collagen biomarkers of type I collagen (reC1M), type III collagen (PRO-C3), type IV collagen (C4G), type VIII collagen (PRO-C8), type XI collagen (PRO-C11), type XVII collagen (PRO-C17) and type VIII collagen (TUM) may be used as potential non-invasive biomarkers.

**Methods:**

We measured the seven biomarkers of collagen turnover in sera of 72 patients with BTC at baseline and after first and second chemotherapy cycle (CTX). Markers were also assessed in sera of 50 healthy controls and compared to levels of patients at baseline. The diagnostic and prognostic value of the markers was evaluated for overall survival (OS) and progression-free survival (PFS).

**Results:**

Patients had a median age of 65 years (IQR 57–70), while healthy controls were younger, with a median age of 46 years (IQR 38–54). The majority of patients (62%) were diagnosed with intrahepatic bile duct adenocarcinoma. Except C4G, all collagen turnover markers were significantly (*p* < 0.001) increased in serum from patients with BTC compared to healthy controls. PRO-C3 was the best marker to discriminate between patients with BTC and controls, reaching an area under a receiver operating characteristic (AUROC) of 0.98 (95% CI 0.95; 0.99) with a sensitivity (92%) and specificity (94%) balanced cutoff of 77.3 ng/ml. Patients with high levels (cohort separated by median split) of PRO-C8 (HR 2.85, 95% CI 1.42; 5.73) followed by C3M (HR 2.33, 95% CI 1.2; 4.5), PRO-C3 (HR 3.09, 95% CI 1.5; 6.36) and CA 19–9 (HR 2.52, 95% CI 1.37; 4.64) as reference biomarker had a shorter OS. Notably, only the novel marker PRO-C8 was also predictive of PFS (HR 3.26, 95% CI 1.53; 6.95). Associations with survival outcomes remained significant after adjusting for relevant risk factors (CA 19–9 and CEA at baseline, age, presence of metastases, weight, height and gender).

**Conclusion:**

The collagen turnover markers PRO-C8, C3M, PRO-C3 and the established biomarker CA 19–9 were prognostic for OS in patients with BTC while only PRO-C8 was also predictive for PFS. PRO-C3 showed the best diagnostic performance to discriminate between patients with BTC and controls.

**Trial registration:**

*Trial registration number and date of registration*

NCT00661830 (NCT number)

15 April 2008

*Trial registry*

The complete registry can found under:

https://clinicaltrials.gov/study/NCT00661830?tab=table#administrative-information (last accessed 01/2025)

*Principal investigator and study sponsor*

Markus Moehler, MD

Johannes Gutenberg University Mainz

**Supplementary Information:**

The online version contains supplementary material available at 10.1186/s12876-025-03645-0.

## Introduction

Biliary tract cancer (BTC) derives from epithelial lining of the biliary tract and is categorized in intrahepatic bile duct, extrahepatic bile duct, gall bladder, and ampulla of Vater [1]. BTC ranges on place 5 of the most common gastrointestinal cancers and carries a highly lethality with a 5-year survival rate of < 20% across all subtypes [2, 3].

The only curative therapeutic approach is surgery when patients are in an early stage. However, a significant proportion of patients presents in an advanced stage, where systemic therapy remains the only available therapeutic option [4]. Despite recent benefits due the addition of immunotherapy to chemotherapy regimens, the prognosis is still dismal. The median overall survival (OS) is 12 months for patients in good performance status (PS), who receive standard first-line treatment with cisplatin and gemcitabine. While the addition of durvalumab, a monoclonal antibody that blocks the interaction of programmed cell death ligand 1 (PD-L1) with PD-1 (CD279), improved the 24-month OS rate to 24.9% (95% CI, 17.9 to 32.5) versus 10.4% (95% CI, 4.7 to 18.8) for placebo, many patients are still not benefitting from therapy [5]. Thus, biomarkers with a high sensitivity and specificity to guide diagnosis, prognosis and therapy are needed in the clinic.

Carbohydrate antigen 19–9 (CA 19–9) and carcinoembryonic antigen (CEA) are the most studied tumor markers in BTC and are increased in 85% and 40% of patients, respectively, compared to healthy controls [6]. Both markers are also prognostic for OS, while in one study CEA was a stronger prognostic biomarker for long-term survival after tumor resection than CA 19–9 [7]. However, also a wide variation in sensitivity (50–90%) and specificity (54–98%) for both serum biomarkers have been reported [8]. In addition, 10% of individuals do not express the Lewis antigen and, therefore, do not produce CA 19–9. Another limitation of these biomarkers is that tumor cells occasionally lose the ability to express tumor markers [9]. Given these limitations, there is a growing need to identify more reliable biomarkers that reflect the underlying biology of BTC and provide greater diagnostic and prognostic accuracy.

**Fig. 1:**
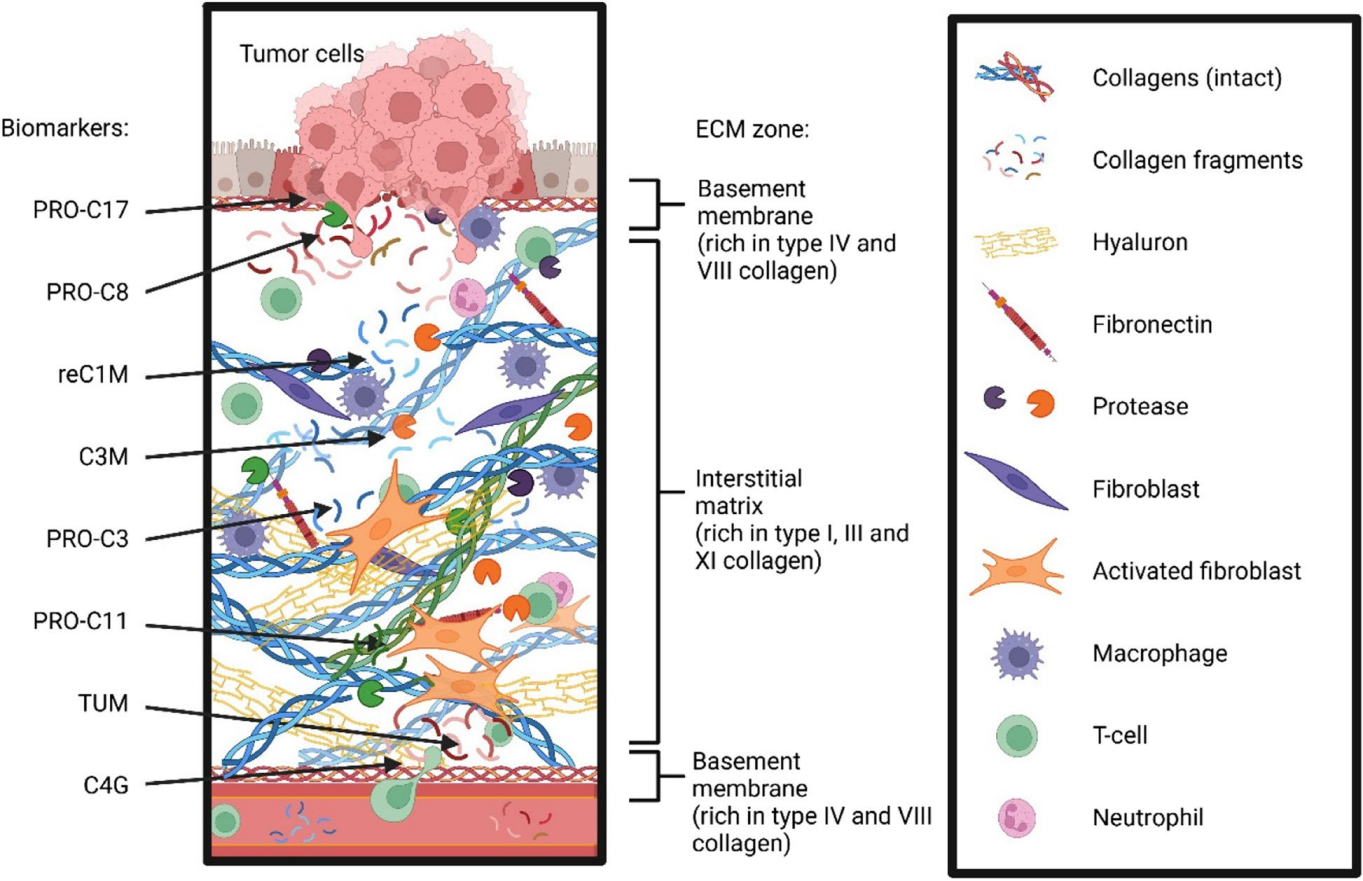
Schematic overview of the distribution of different collagens in the tumor microenvironment (TME) of biliary tract cancer (BTC). Collagen remodeling is driven by various proteases such as matrix metalloproteases (MMPs) produced by tumor cells and macrophages as well as serine proteases, collagen degrading enzymes, such as neutrophil elastase produced by neutrophils, or granzyme B (GzB) produced primarily by T-cells and NK-cells. These remodeling processes release collagen turnover markers into circulation where they can be quantified non-invasively as biomarkers in a liquid biopsy. The collagen turnover markers applied in this study and their respective compartment and origin in the tumor microenvironment include: reC1M (a fragment generated by MMP-degraded type I collagen), C3M (a fragment generated by MMP-degraded type III collagen), PRO-C3 (the aminoterminal pro-peptide of type III procollagen released as part of synthesis and fibrogenesis), TUM (Tumstatin, NC1 domain of type VIII collagen), C4G (a fragment generated by GzB-degraded type IV collagen), PRO-C8 (Vastatin, NC1 domain of type VIII collagen) PRO-C11 (the aminoterminal pro-peptide of type XI collagen released as part of synthesis and fibrogenesis) and PRO-C17 (the type XVII collagen ectodomain)

In the last decades, the tumor microenvironment (TME) and here especially the extracellular matrix (ECM) gained focus in the search of novel biomarkers as it has implications for immunotherapy, which became an essential component in oncologic therapy [10]. The ECM is heavily altered in BTC and characterized by a desmoplastic tumor stroma, consisting among others of 28 different collagens [11]. Desmoplastic tumor stroma is a dense, fibrous tissue that forms around tumors, promoting tumor growth and metastasis. This pathological feature is characteristic of BTC and pancreatic cancer but is also observed in other adenocarcinomas [12–14]. The collagens of the ECM are unique and have specialized structural, biochemical, and biomechanical roles in the tissue [11]. Type IV collagen and type VIII collagen are primarily found in the basement membrane zone and are important for epithelial cell polarity and angiogenesis. Type I collagen, type III collagen and type XI collagen are found in the interstitial matrix zone produced by fibroblasts in the underlying stroma. Type XVII collagen is anchored in the membrane of epithelial cells and directly connects the epithelium to the basement membrane. Under physiological conditions, synthesis and degradation of the different collagens is highly regulated for tissue homeostasis, while a desmoplastic tumor stroma occurs due to excessive collagen turnover and remodeling. The collagen remodeling and degradation is driven by various proteases such as matrix metalloproteases (MMPs) produced for example by tumor cells and macrophages as well as serine proteases such as neutrophil elastase, or granzyme B (GzB) produced primarily by T-cells and NK-cells (Fig. 1). All this altered activity (increased collagen synthesis and degradation) results in loss of tissue organization, which impedes correct cellular behavior and consequently fosters disease progression. specific collagen fragments are released into circulation during these processes and can be measured non-invasively in a liquid biopsy as biomarkers [15, 16]. Collagen biomarkers have already proven their value to predict outcomes in patients with solid cancer, while data for patients with BTC is scarce [17–20].

In this explanatory study, we investigated non-invasive biomarkers of type I collagen, type III collagen, type IV collagen, type VIII collagen, type XI collagen and type XVII collagen remodeling in serum from patients with BTC, which have shown in part a diagnostic and prognostic value in patients with this disease before [17]. In detail, we measured reC1M (a fragment generated by MMP-degraded type I collagen) [21–23], C3M (a fragment generated by MMP-degraded type III collagen), PRO-C3 (the pro-peptide of type III procollagen released as part of synthesis and fibrogenesis), TUM (Tumstatin, noncollagenous carboxyterminal NC1 domain of type VIII collagen) [24], C4G (a fragment generated by GzB-degraded type IV collagen), PRO-C8 (Vastatin, NC1 domain of type VIII collagen) [25], PRO-C11 (the aminoterminal pro-peptide of type XI procollagen released as part of synthesis and fibrogenesis) [26], PRO-C17 (the type XVII collagen ectodomain) [27], and evaluated the diagnostic and prognostic performance in a well-characterized study cohort of patients with BTC, who were recruited in a multicenter, randomized clinical trial.

## Results

### Baseline

Seventy-two patients with BTC and 50 healthy controls were included in the cohort (Table 1). Healthy controls were younger and had a median age of 46 years (IQR 38; 54) than the included patients with a median age of 65 years (IQR 57; 70). Sex of patients and healthy controls was equally balanced (men 56% versus 52%).

**Table 1:** Baseline characteristics of included patients with biliary tract cancer and healthy controls

		**Tumor patients**	**Healthy controls**	
**Total, n (%)**		72 (100)	50 (100)	
**Age, median (IQR)**		65 years (57; 70)	46 years (38; 54)	
**Gender, n (%)**	male	40 (56)	26 (52)	
**Diagnosis, n (%)**	Adenocarcinoma of intrahepatic bile ducts	45 (62)		
	With intrahepatic metastases	20 (28)		
	Adenocarcinoma of gall bladder	7 (10)		
**BMI, n (%)**	BMI < 25	35 (50)		
	BMI 25–30	24 (33)		
	BMI > 30	10 (13)		
	unknown	3 (4)		
**UICC stage, n (%)**	1	2 (3)		
	2	2 (3)		
	3	28 (38)		
	4	38 (53)		
	unknown	2(3)		
**G, n (%)**	G1	1 (1)		
	G2	42 (59)		
	G3	23 (33)		
	G4	1 (1)		
	unknown	5 (6)		
**Overall survival, median (IQR)**		312 days (154; 474)		
**Progression-free survival, median (IQR)**		106 days (50; 245)		

*BMI* body mass index, *G* histological grading, *UICC* Union for International Cancer Control

The majority of patients were diagnosed with adenocarcinoma of intrahepatic bile ducts (62%), followed by hepatic metastases (28%) and adenocarcinoma of the gall bladder (10%). Most patients were in an advanced tumor stage (91%, Union for International Cancer Control (UICC) stage III and IV). Median OS and progression-free survival (PFS) were 312 days (IQR 154; 474) and 106 days (IQR 50; 245), respectively.

### Collagen turnover markers are elevated in serum of patients with biliary cancer

**Fig. 2:**
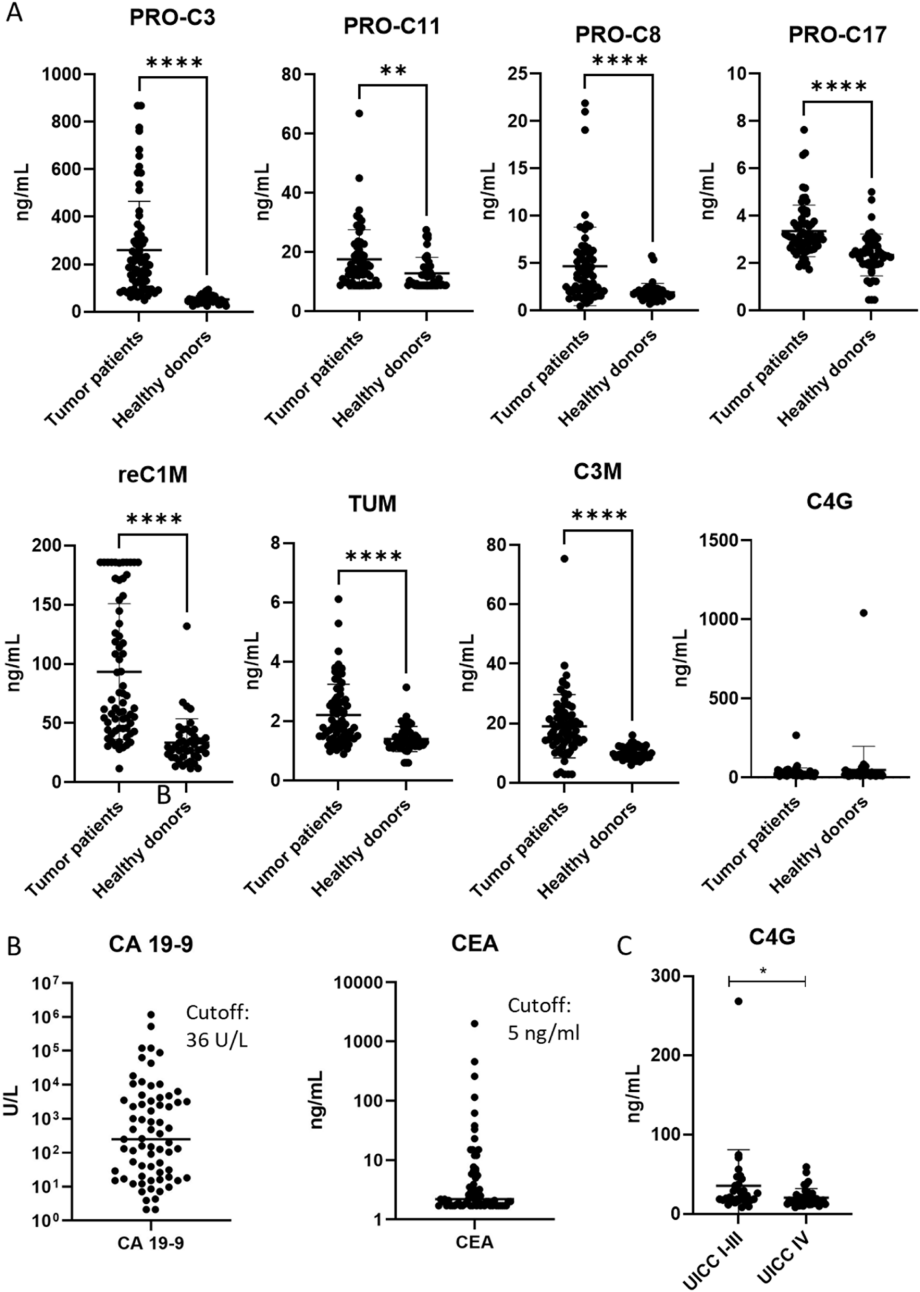
**A** Quantification of the collagen turnover markers in sera of patients with biliary tract cancer (BTC) and healthy controls at baseline. **B** Quantification of the established tumor markers CA 19–9 and CEA in sera of patients with BTC plus the corresponding established cutoffs at baseline. **C** Comparison of C4G levels of patients with BTC, who were stratified for Union for International Cancer Control (UICC) stage I-III versus IV (*,**,****p < 0.05, 0.001, 0.0001)

A panel of eight biomarkers were quantified in sera of patients with BTC and healthy controls. Seven of eight determined markers PRO-C3, PRO-C8, PRO-C11, PRO-C17, reC1M, C3M and TUM were significantly (*p* < 0.0001) increased in patients compared to controls (Fig. 2A). Only, the marker C4G showed no significant difference. Levels of the established tumor markers CA 19–9 and CEA were also quantified as reference (Fig. 2B). Here, 69% patients in our cohort for CA 19–9 and 24% for CEA were above the established cutoffs. Next, we tested if the markers may also reflect the stage of the disease. Overall, the collagen turnover markers did not differ between patients with local and locally advanced disease (UICC stage I-III) versus advanced disease (UICC stage IV). Only the levels of C4G were lower in patients in UICC stage IV (Fig. 2C). There was no difference in biomarker levels when BTC patients were stratified by histological tumor grade: G1/2 (well- and moderately differentiated) versus G3/4 (poorly differentiated and undifferentiated). This is illustrated in Supporting Fig. 1.

### Quantification of collagen turnover markers after first and second chemotherapy cycle

**Fig. 3:**
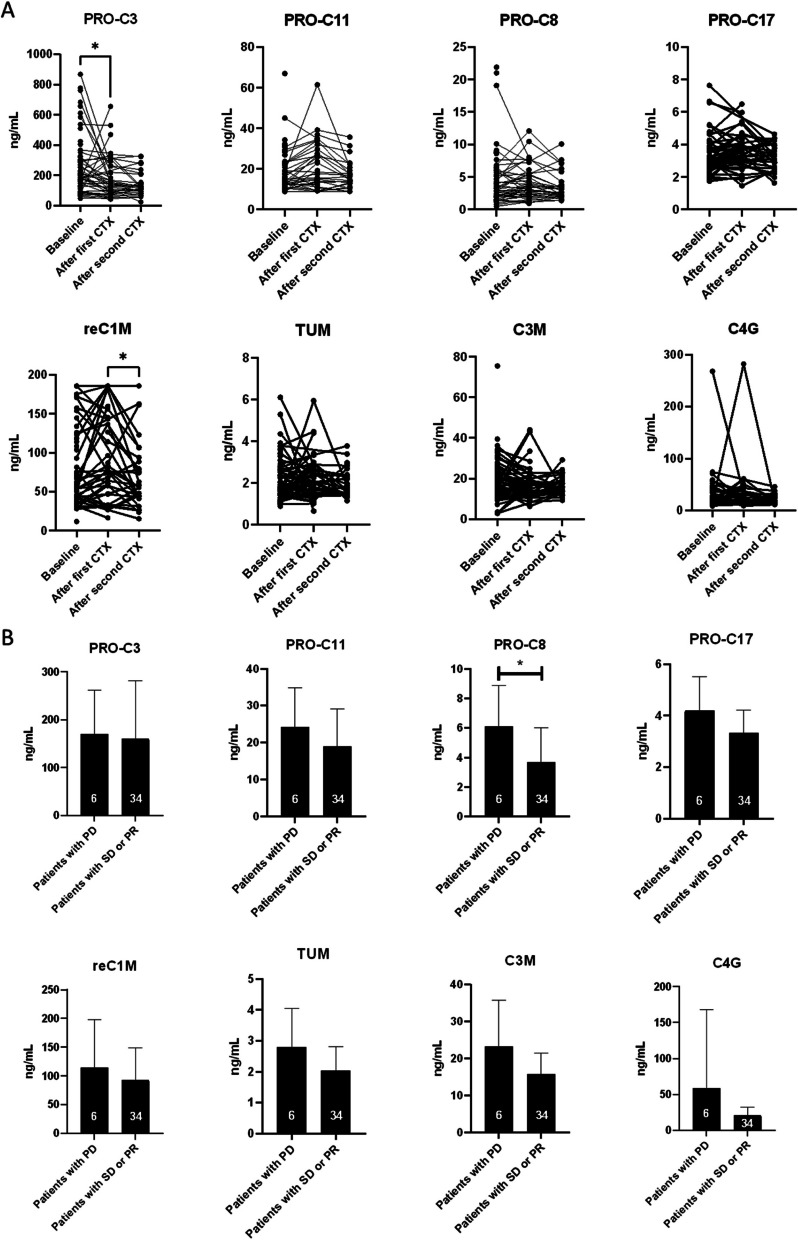
**A** Comparison of collagen turnover marker levels in patients with biliary tract cancer (BTC) at baseline compared to after first and second chemotherapy cycles (CTX). **B** Comparison of collagen turnover marker levels of patients with progressive disease (PD) versus patients with stable disease (SD) or partial response (PR) after first chemotherapy cycle (*p < 0.05, 0.001, 0.0001; for 2 B n = are shown on the bars)

Next, we tested whether levels of collagen turnover markers were affected by the chemotherapy. There was no clear trend that the collagen turnover markers were down- or upregulated by the applied regime as none of the markers consistently increased or decreased after the first and second chemotherapy cycle (Fig. 3A). Only PRO-C3 and reC1M decreased after the first and second cycle, respectively. For PRO-C3, this was particularly evident in patients with high levels at baseline.

There is plausibility that only (partial) responders may have shown deregulated levels of the collagen turnover markers. Therefore, patients were stratified in progressive disease (PD) versus stable disease (SD) and partial response (PR) after the first chemotherapy cycle. Here, PRO-C8 was significantly downregulated in patients with SD and PR (Fig. 3B).

**Fig. 4:**
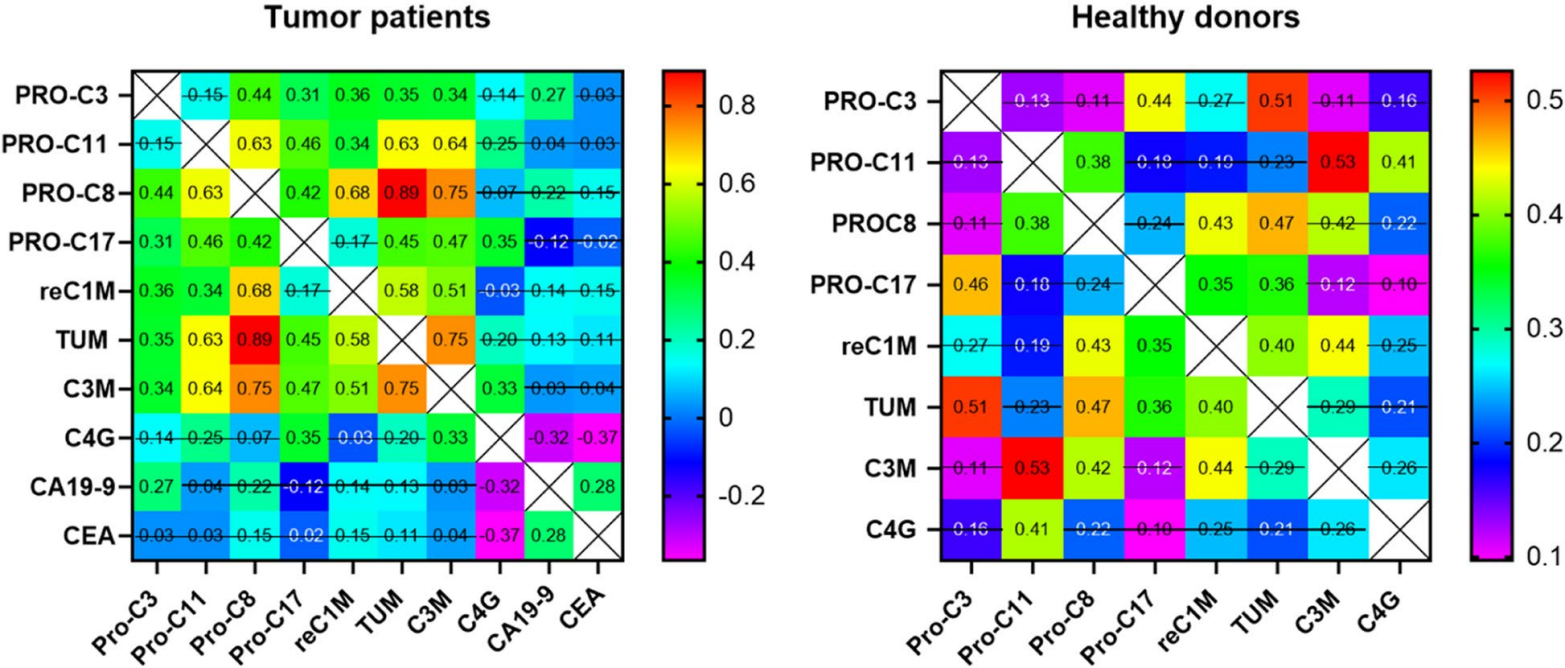
Correlation matrix of the collagen turnover markers in tumor patients and healthy controls at baseline of the study (*p*-values > 0.05 are crossed)

Since all of the collagen turnover markers derive from certain collagen fragments and are involved in remodeling of particular subdomains of the ECM, we tested to which extent the markers correlate with each other. Here, almost all assessed markers correlated with each other reaching a Spearman’s correlation coefficient up to 0.89 for TUM with PRO-C8. Correlations were less pronounced in healthy controls. Interestingly, C4G together with the established biomarkers CA 19–9 and CEA markers showed only minor correlations in the patient cohort (Fig. 4).

### Diagnostic performance of the collagen turnover markers

The diagnostic performance of the collagen turnover markers to discriminate between patients and healthy controls was assessed by AUROC analysis (Table 2). In addition, sensitivity and specificity balanced cut-offs (Youden index) were calculated for the markers. Here, PRO-C3 had the highest diagnostic value with an AUROC of 0.98 with a cutoff of 77.3 ng/ml, giving a sensitivity of 0.92 and a specificity of 0.94, while C4G had the lowest diagnostic performance (AUROC of 0.50 and a cutoff of 18.1, 0.57 sensitivity, 0.50 specificity).

**Table 2:** Diagnostic performance of the collagen turnover markers to discriminate between tumor patients and healthy controls by AUROC analysis

		**AUROC**	**Cutoff (ng/mL)**	**Sensitivity**	**Specificity**
**1**	**PRO-C3**	0.98 (95% CI 0.95; 0.99)	77.3	0.92	0.94
**2**	**C3M**	0.85 (95% CI 0.78; 0.93)	13.8	0.73	0.98
**3**	**reC1M**	0.88 (95% CI 0.82; 0.94)	45.4	0.76	0.86
**4**	**PRO-C17**	0.80 (95% CI 0.72; 0.88)	2.5	0.85	0.68
**5**	**PRO-C8**	0.78 (95% CI 0.7; 0.87)	2.5	0.63	0.93
**6**	**TUM**	0.77 (95% CI 0.69; 0.85)	1.7	0.61	0.84
**7**	**PRO-C11**	0.67 (95% CI 0.57; 0.78)	12.3	0.68	0.64
**8**	**C4G**	0.50 (95% CI 0.40; 0.61)	18.1	0.57	0.50

*CI* confidence interval

### Collagen turnover markers predict overall survival

**Fig. 5:**
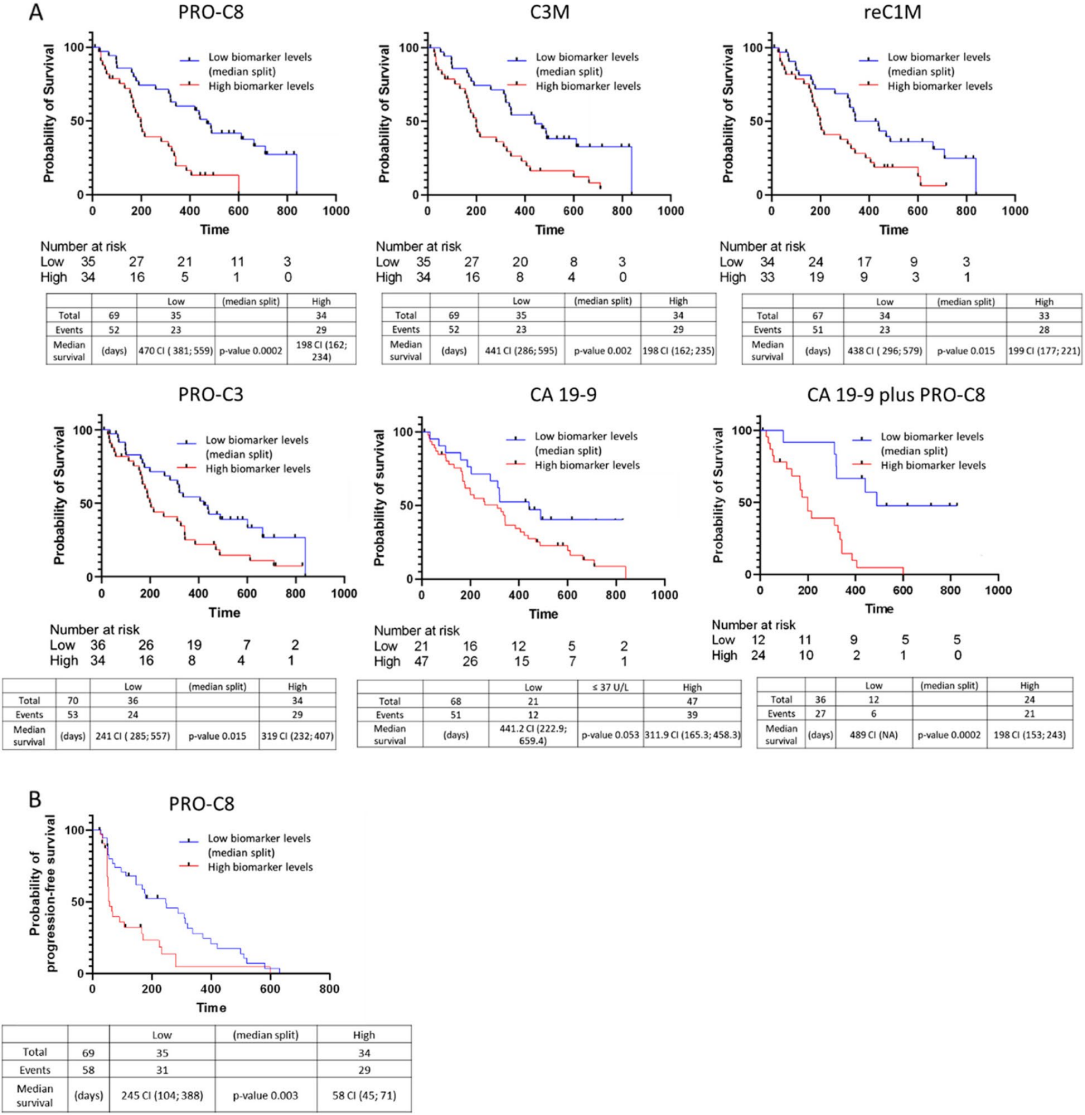
**A** Kaplan–Meier survival plots show that levels of PRO-C8, C3M, reC1M, PRO-C3, CA 19–9 and combination of CA 19–9 plus PRO-C8 predict OS in patients with biliary tract cancer (BTC). **B** High levels of PRO-C8 predict also PFS of the patients (the cohort was separated into low and high levels of collagen turnover markers by median split at baseline)

Since the biomarkers were increased in tumor patients, we evaluated their prognostic value for OS and PFS in our cohort. Therefore, patients were dichotomized according to their marker levels into a “low” and “high” group by median split. Based on Kaplan Meier analysis, PRO-C8 showed the best prognostic value (log-rank, *p*-value = 0.0002) for OS in the cohort, followed by C3M, reC1M, PRO-C3 and CA 19–9. Patients with low PRO-C8 levels had a median OS of 470 days (95% CI 470; 559) compared to 198 days (95% CI 162; 234) of patients with high PRO-C8 levels. As PRO-C8 showed the best predictive performance for OS, we combined PRO-C8 (high) with the established biomarker CA 19–9 (high). The prognostic performance could not be improved by the combination of the markers, reaching an equal log-rank *p*-value of 0.0002 as PRO-C8 alone (Fig. 5A). In longitudinal analysis, patients who responded to CTX with a decrease of biomarker levels (< 20% reduction from baseline to after first CTX) had no longer OS compared to non-responders (Supporting Fig. 2).

Furthermore, only PRO-C8 showed a prognostic value for PFS. Similar to OS, the patients with low levels of PRO-C8 had a longer median PFS of 245 days (95% CI 104; 388) versus 58 days (95% CI 45; 71) of patients with high levels (log-rank, *p* = 0.003, Fig. 5B).

The ability of C3M, reC1M and PRO-C3 to predict OS and of PRO-C8 to predict OS and PFS was then investigated with a multivariate Cox proportional-hazards model stratified by age, gender, CEA, CA19-9, biometric parameters (height, weight) and presence of metastases. Except reC1M, all of the collagen turnover markers remained independently predictive for OS (and PFS in case for PRO-C8) (Table 3). The analysis was also done with the collagen turnover markers as continuous variable (Supporting Table 1). Here, all markers remained prognostic.

**Table 3:** Multivariate Cox proportional-hazards models of the collagen turnover markers PRO-C8, C3M, PRO-C3, and CA19-9 at baseline as dichotomized variable (median split) to predict overall survival (OS) and PFS

**PRO-C8 (OS)**	***p*-value**	**Hazard ratio**	**95% confidence interval**	
			**Lower**	**Upper**
Age	0.002	1.054	01. Feb	1.088
Gender (male 0, female 1)	0.123	0.573	0.283	1.163
CA19-9	0.169	1	1	1
Height	0.532	0.995	0.978	1.012
Weight	0.979	1	0.97	1.032
CEA	0.931	1	0.999	1.001
Metastases (no 0, yes 1)	0.31	1.465	0.701	3.062
***PRO-C8***	***0.003***	***2.847***	***1.416***	***5.725***
**PRO-C8 (PFS)**	***p*-value**	**Hazard ratio**	**95% confidence interval**	
			**Lower**	**Upper**
Age	0.039	1.036	1.002	1.071
Gender (male 0. female 1)	0.959	0.981	0.472	2.038
CA19-9	0.851	1	1	1
Height	0.677	1.004	0.986	1.021
Weight	0.865	0.997	0.966	1.029
CEA	0.752	1	0.999	1.001
Metastases (no 0, yes 1)	0.612	1.234	0.548	2.778
***Pro-C8***	***0.002***	***3.262***	***1.531***	***6.952***
**C3M (OS)**	***p*-value**	**Hazard ratio**	**95% confidence interval**	
			**Lower**	**Upper**
Age	0	1.059	1.026	1.093
Gender (male 0, female 1)	0.593	0.828	0.415	1.654
CA19-9	0.028	1	1	1
Height	0.607	0.995	0.978	1.013
Weight	0.881	0.998	0.967	1.029
CEA	0.849	1	0.999	1.001
Metastases (no 0, yes 1)	0.235	1.546	0.754	3.169
***C3M***	***0.012***	***2.325***	***1.201***	***4.5***
**PRO-C3 (OS)**	***p*-value**	**Hazard ratio**	**95% confidence interval**	
			**Lower**	**Upper**
Age	0.002	1.049	1.018	1.08
Gender (male 0, female 1)	0.637	0.844	0.417	1.707
CA19-9	0.505	1	1	1
Height	0.582	0.995	0.979	1.012
Weight	0.588	0.992	0.964	1.021
CEA	0.967	1	0.999	1.001
Metastases (no 0, yes 1)	0.009	3.196	1.319	7.740
***PRO-C3***	***0.002***	***3.092***	***1.503***	***6.364***
**CA19-9** **(OS)**	***p*-value**	**Hazard ratio**	**95% confidence interval**	
			**Lower**	**Upper**
Age	0.001	1.045	1.018	1.074
Gender (male 0, female 1)	0.373	0.743	0.387	1.428
Height	0.365	0.993	0.978	1.008
Weight	0.856	0.998	0.972	1.024
CEA	0.938	1	0.999	1.001
Metastases (no 0, yes 1)	0.028	2.287	1.095	4.776
***CA19-9***	***0.003***	***2.521***	***1.37***	***4.637***
**reC1M (OS)**	***p*-value**	**Hazard ratio**	**95% confidence interval**	
			**Lower**	**Upper**
Age	< 0.001	1.071	1.032	1.112
Gender (male 0, female 1)	0.072	0.525	0.261	1.059
CA19-9	0.02	1	1	1
Height	0.539	0.994	0.977	1.012
Weight	0.832	0.997	0.966	1.029
CEA	0.886	1	0.999	1.001
Metastases (no 0, yes 1)	0.137	1.768	0.834	3.747
***reC1M***	***0.16***	***1.642***	***0.822***	***3.28***

*OS* overall survival, *PFS* progression-free survival

## Discussion

Collagen markers, which reflect extracellular matrix remodeling, are increasingly recognized as important in biliary tract cancer (BTC). Elevated collagen deposition and remodeling, often driven by tumor-stromal interactions, contribute to a desmoplastic tumor microenvironment that hinders drug delivery and promotes resistance to chemotherapy. Specific collagen biomarkers, which can be assessed in liquid biopsy, are associated with aggressive tumor behavior and poor prognosis in BTC. [11, 28]. In our study, we found that the serum collagen turnover markers related to stroma turnover of type I collagen (reC1M), type III collagen (PRO-C3, C3M), type VIII collagen (PRO-C8, TUM), type XI collagen (PRO-C11), type XVII collagen (PRO-C17) were elevated in patients with BTC compared to healthy controls.

PRO-C3 showed the best diagnostic performance to discriminate between patients with BTC and healthy individuals, reaching an AUROC of 0.98 (95% CI 0.95; 0.99). When using a cut-off of 77.3 ng/mL a sensitivity of 92% and specificity of 94% was reached. Another interesting question was whether the markers may reflect the disease stage (UICC) in patients with BTC. Only C4G was significantly deregulated, when comparing early (UICC stage I, II) with advanced stage (UICC stage III, IV), where C4G was mildly upregulated in early stages of disease. The lack of difference between most markers is in line with previously published data from Christensen et al., from a large cohort, consisting of 269 patients with BTC and 49 patients with benign biliary tract diseases [17]. Notably, ProC8, the key predictive and potentially therapy response marker, was not assessed in this prior cohort, which also relied only on patients’ baseline sera. Further, PRO-C3, C3M and PRO-C6 biomarker levels did not differ between BTC patients with resectable disease, locally advanced disease, and metastatic disease, while levels for C4G were significantly lower in patients with resectable disease compared to locally advanced disease and metastatic disease. However, the C4G finding lacks plausibility considering that C4G was the only marker, which was not elevated in patients with BTC versus healthy controls. However, the fact that type III collagen (C3M and PRO-C3) and type XI collagen (PRO-C11) biomarkers were found to be elevated across disease stages highlights the importance of remodeling of the interstitial matrix zone of the collagen produced by fibroblasts in the underlying stroma at all stages of disease in BTC. Similar to the present findings, Christensen et al. have evaluated C3M, PRO-C3 and PRO-C11 for association with OS and found that PRO-C3 was predictive for OS in patients with first- and second line therapy, while PRO-C11 did not reach significance and C3M was only significant in patients with first-line therapy, being in line with the findings in our analysis for OS.

None of the markers was constantly downregulated after first and second CTX compared to baseline or to biomarker levels after first CTX, respectively, while only levels of reC1M decreased after the second CTX, which may be attributed to a selection bias of the surviving patients. There is plausibility that only biomarker levels of patients who responded to CTX (SD and PR) would have decreased. Here, levels of PRO-C8 were lower in patients who were in SD or PR compared to patients with PD. Thus, PRO-C8 may provide a diagnostic value to evaluate therapeutic response in patients with BTC.

One of the most vital tasks of biomarkers in oncology is their prognostic value to predict outcomes or interim consequences [29]. High serum levels of biomarkers related to turnover of type I, III and VIII collagens (reC1M, C3M, PRO-C3 and PRO-C8) were prognostic for OS of patients with BTC. Among these, PRO-C8 was the strongest predictor for OS (*p*-value = 0.0002) and the only marker, which could also predict PFS (*p*-value = 0.003). Beside the collagen turnover markers, the established biomarker CA 19–9 was prognostic for OS. However, the combination of PRO-C8 and CA 19–9 could not further improve prognostic value for OS, suggesting the use of PRO-C8 as standalone biomarker of e.g. CA 19–9 negative tumors. The associations of the assessed biomarkers with OS and PFS remained significant after adjusting for risk factors, which may affect survival of tumor patients (age, gender, biometric parameters, levels of established tumor markers CA 19–9 and CEA, presence of metastases).

Beside BTC, increased serum levels of PRO-C3 have been associated with poor survival of patients with other solid cancers e.g. pancreatic cancer, breast cancer, and melanoma [18, 20, 30, 31]. PRO-C3 is released during fibrogenesis and is associated with cancer-associated fibroblast (CAF) activity [32]. CAFs shape the TME in favor of the tumor and support cancer development, growth and metastasis by a multitude of mechanisms, including ECM remodeling and angiogenesis [33]. High abundance of CAFs was associated with poor survival in patients with intrahepatic cholangiocarcinoma, suggesting that assessment of CAF activity by the PRO-C3 biomarker may provide an additional prognostic value. Beside malignant disease, the prognostic performance of PRO-C3 could also be shown in patients with advanced fibrotic liver diseases of all etiologies. A 2-fold increase in PRO-C3 in patients with rapid fibrosis progression was associated with 2.7-fold increased hazard of liver-related morbidity and mortality [34]. The prognostic and diagnostic utility of PRO-C3 is also evident in cholestatic liver disease and autoimmune hepatitis [35], where PRO-C3 correlated with liver stiffness and showed a good diagnostic performance to discriminate between advanced and non-advanced fibrotic liver disease. In these patients, levels of PRO-C3 responded also to anticholestatic treatment with ursodeoxycholic acid. In another study, the collagen turnover markers PRO-C3, PRO-C8 and C4M have also been studied in patients with acute-on-chronic liver failure (ACLF), which is a devastating exacerbation in patients with cirrhosis and an acute onset of decompensation of the chronically damaged liver [36]. All biomarkers were significantly elevated in patients with ACLF compared to healthy controls or patients with cirrhosis without ACLF, while none of them could predict 28- and 90-day mortality. This set of biomarkers have been evaluated also in patients with mild to modest metabolic dysfunction-associated steatotic liver disease (MASLD) and severe obesity, undergoing bariatric surgery [37]. Twelve months after surgery, C4M and PRO-C8 decreased by 24% and 44%, respectively, while PRO-C3 remained unchanged. Beside liver disease, PRO-C8 was transiently elevated during acute hemarthrosis in hemophilic arthropathy, reflecting the relevance of this biomarker also for measuring basement membrane turnover in extrahepatic disease [38]. Interestingly, type VIII collagen was recently found to be produced almost exclusively by CAFs from pancreatic cancer patients and not by pancreatic fibroblast from non-malignant tissue, indicating that PRO-C8, similar to PRO-C3, could be a marker of CAF activity [39]. To our knowledge, the present study is the first to show PRO-C8 as a prognostic biomarker in BTC, which is further pointing to the importance of basement membrane integrity (altered type VIII collagen turnover) as an essential component for outcome of patients with BTC.

Due to the retrospective nature of the study design, some limitations have to be acknowledged. For the included patients, there was no clinical information documented neither for chronic parenchymal damage of organs (e.g. liver cirrhosis/fibrosis, lung fibrosis, scleroderma nor chronic kidney disease), which could have influenced levels of the collagen turnover markers. The included healthy controls were younger than the tumor patients (65 years (IQR 57; 70) vs. 46 years (IQR 38; 54)), which may complicate the comparison as biomarkers can increase with age. We cannot provide additional healthy donor characteristics as only sex and age of the donors were reported by the blood bank due data privacy regulations.

Furthermore, given the extensive number of tests conducted for group comparisons in this study, the p-values should be interpreted with caution. As most analyses involve pairwise group comparisons without adjustments for multiple testing, there is an increased risk of false-positive results. Further, validation of through appropriately powered studies and rigorous correction methods are needed to confirm the biomarkers’ predictive value in external cohorts.

Taken together, the collagen III derived biomarkers PRO-C3 and C3M had the best diagnostic performance to discriminate between patients with BTC and healthy individuals and were predictive for OS in patients with BTC as shown before in a larger cohort [17]. Collagen III-derived biomarkers hold promise in BTC patients, independent of liver parenchymal status, as they may reflect tumor fibrosis rather than liver fibrosis [17]. This distinction is clinically significant, given that approximately 30% of BTC patients have cirrhosis, which is a major risk factor for the disease [40].

Beside PRO-C3, we could demonstrate for the first time both diagnostic and prognostic accuracy of the collagen fragments reC1M (collagen I) and especially PRO-C8 (collagen VIII) in patients with BTC. PRO-C8 may provide also a diagnostic benefit to determine response to therapy.

Further studies with external validation cohorts are needed to evaluate the diagnostic and prognostic value of collagen III and VIII fragments in patients with BTC and other solid tumors characterized by desmoplastic stroma, such as pancreatic cancer.

## Material methods

### Patient and healthy donor samples

Serum samples from 72 patients with BTC were obtained from the GEMSO study “Gemcitabine plus sorafenib versus gemcitabine alone in advanced biliary tract cancer: A double-blind placebo-controlled multicenter phase II AIO study with biomarker and serum program” from 2008 to 2010 from eleven German hospitals [41]. Sorafenib is an anti-angiogenic small molecule drug and is not considered standard of care for biliary tract cancer (BTC). Explicit details of inclusion and exclusion criteria as well as study design are published by Moehler et al. [41]. Serum samples were collected at baseline and after first and second chemotherapy cycle. Chemotherapy consisted of Gemcitabine (1000 mg/m2), which was administered at day 1, 8, 15, 22, 29, 36, 43 of the first cycle (8 weeks’ duration) and at day 1, 8 and 15 of all subsequent cycles (4 weeks’ duration). Sorafenib (400 mg) or placebo tablets were administered twice daily.

Serum samples of healthy controls were obtained from the blood donor center at the University Medical Center of the Johannes Gutenberg-University, while only age and sex of the donors were reported in line with the German data privacy act.

### Quantification of the collagen turnover markers by ELISA

All the collagen turnover biomarkers were measured by competitive ELISA according to the manufacturer’s instructions (Nordic Bioscience, Herlev, Denmark). The technical evaluation and assay development have been described elsewhere for each assay: reC1M (cat.no. 1000AG01 (NordicC1M™) [23], a fragment generated by MMP-degraded type I collagen), C3M (cat.no. 1200AG01 (NordicC3M™) [22], a fragment generated by MMP-degraded type III collagen), PRO-C3 (cat.no. 1700AF01 (NordicPRO-C3™) [21], the pro-peptide of type III collagen released as part of synthesis and fibrogenesis), TUM (cat.no. 4800AG01 (NordicTUM™) [24], Tumstatin, NC1 domain of type VIII collagen), C4G (cat.no. 1027CE01 (NordicC4G™), a fragment generated by GzB-degraded type IV collagen), PRO-C8 (cat.no. 0106AE01 (NordicPRO-C8™) [25], Vastatin, NC1 domain of type VIII collagen) PRO-C11 (cat.no. 1020AE01 (NordicPRO-C11™) [26], the pro-peptide of type XI collagen released as part of synthesis and fibrogenesis), PRO-C17 (cat.no. 1084AD01 (NordicPRO-C17™) [27], the type XVII collagen ectodomain). In brief, the general ELISA procedures were as follows: a 96-well streptavidin-coated microtiter plate was coated with a biotinylated peptide dissolved in assay buffer and incubation for 30 min at 20°C in darkness. The plates were then subsequently washed five times in washing buffer (20 mM Tris, 50 mM NaCl, pH 7.2). Then, 20 μl of calibrator peptide, quality control samples, or serum samples was added in duplicates to appropriate wells followed by addition of 100 μl of horse-radish peroxidase monoclonal antibody solution in assay buffer. The plate was incubated for 1–20 h at 4°C or 20°C depending on the assay and followed by an additional washing step. Finally, 100 μl of tetramethylbenzinidine (Kem-En-Tec, Taastrup, Denmark; cat. #438OH) was added before incubating 15 min at 20°C, followed by 100 μl of stopping solution (1% H2SO4). The plates were shaking with 300 rpm during all incubation steps. Finally, the optical density was measured at 450 nm with 650 nm as reference and a four-parametric mathematical fit model was used to plot a calibration curve.

### Statistics

Group comparisons for collagen turnover marker levels in patients with BTC versus healthy controls and after each CTX in patients with BTC were done by Mann–Whitney U-test. Correlation analyses were conducted using Spearman's rank correlation. The complete data analysis was exploratory. Hence, no adjustments for multiple testing were performed. The diagnostic accuracy of the markers was assessed by calculating the area under the receiver operating characteristics curve (AUROC) and evaluating the ability to discriminate individual cancer types from healthy controls. Sensitivity and specificity balanced cutoffs were calculated by the Youden Index. Prognostic value of the markers for OS /PFS was evaluated by comparing patients with low and high levels (median split). Kaplan–Meier curves and log-rank tests were applied to determine differences between the survival curves. Multivariate Cox regression analysis was performed to evaluate the independent prognostic value of the markers after adjusting for age, sex, height, weight, CA 19–9 and CEA levels at baseline and presence of metastases. Significance was considered with *p*-values < 0.05 as it follows: * *p* < 0.05; ** *p* < 0.01; *** *p* < 0.001; **** *p *< 0.0001. Statistical analyses were performed using GraphPad Prism (version 9.5.0 for Windows, GraphPad Software, San Diego, California USA, www.graphpad.com) and IBM SPSS Statistic Version 27.0 (Armonk, NY: IBM Corp).

## Supplementary Information

Supplementary Material 1.

## Data Availability

Data is available on reasonable request and can be provided by the corresponding authors.

## Abbreviations

ACLF: Acute-on-chronic liver failure
AUROC: Area under a receiver operating characteristic
BMI: Body mass index
BTC: Biliary tract cancer
C3M: Fragment generated by MMP-degraded type III collagen
C4G: Collagen biomarkers of type IV collagen
CA 19–9: Carbohydrate antigen 19–9
CAF: Cancer-associated fibroblast
CEA: Carcinoembryonic antigen
CI: Confidence interval
CRC: Collaborative Research Center
CTX: Chemotherapy cycle
DFG: German Research Foundation
ECM: Extracellular matrix
G: Histological grading
GzB: Granzyme B
LITMUS: Liver Investigation on Marker Utility in Steatohepatitis
MASLD: Metabolic dysfunction-associated steatotic liver disease
MMPs: Matrix metalloproteases
OS: Overall survival
PD: Progressive disease
PD-L1: Programmed cell death ligand 1
PFS: Progression-free survival
PR: Partial response
PRO-C11: Collagen biomarkers of type XI collagen
PRO-C17: Collagen biomarkers of type XVII collagen
PRO-C3: Collagen biomarkers of type VIII collagen (NC1 domain)
PRO-C8: Collagen biomarkers of type VIII collagen
PS: Performance status
reC1M: Collagen biomarkers of type I collagen, type I collagen
SD: Stable disease
TME: Tumor microenvironment
TUM: Collagen biomarkers of type VIII collagen (non-collagenous carboxyterminal NC domain)
UICC: Union for International Cancer Control, Union for International Cancer Control
