# Supplementary material for: Collagen turnover biomarkers to predict outcome of patients with biliary cancer
Source: BMC Gastroenterol. 2025 Feb 4;25:53. doi: 10.1186/s12876-025-03645-0 (PMC11792424; doi:10.1186/s12876-025-03645-0)
Supplement: Supplementary file 1 — Supplementary Material 1. [file 12876_2025_3645_MOESM1_ESM.docx]

**Supporting information (SI)**

SI table 1. Multivariate Cox proportional-hazards models of the collagen turnover markers PRO-C8, C3M, PRO-C3, rec1M, PRO-C8 and CA19-9 as continuous variable to predict overall survival (OS) and progression-free survival (PFS)

| PRO-C8 (OS) | p-value | Hazard ratio | 95,0% CI for hazard ratio | |
| --- | --- | --- | --- | --- |
|  |  |  | Lower | Upper |
| Age | 0,001 | 1,057 | 1,024 | 1,092 |
| Gender (male 0, female 1) | 0,191 | 0,627 | 0,312 | 1,261 |
| CA19-9 | 0,062 | 1,000 | 1,000 | 1,000 |
| Height | 0,254 | 0,990 | 0,974 | 1,007 |
| Weight | 0,706 | 1,006 | 0,976 | 1,037 |
| CEA | 0,550 | 1,000 | 0,999 | 1,001 |
| Metastases (no 0, yes 1) | 0,216 | 1,596 | 0,760 | 3,351 |
| ***PRO-C8*** | ***0,002*** | ***1,129*** | ***1,045*** | ***1,220*** |

| PRO-C8 (PFS) | p-value | Hazard ratio | 95,0% CI for hazard ratio | |
| --- | --- | --- | --- | --- |
|  |  |  | Lower | Upper |
| Age | 0,012 | 1,043 | 1,009 | 1,078 |
| Gender (male 0, female 1) | 0,789 | 1,105 | 0,533 | 2,289 |
| CA19-9 Baseline | 0,387 | 1,000 | 1,000 | 1,000 |
| Height | 0,732 | 0,997 | 0,981 | 1,014 |
| Weight | 0,863 | 1,003 | 0,972 | 1,034 |
| CEA at baseline | 0,617 | 1,000 | 0,999 | 1,001 |
| Metastases (no 0, yes 1) | 0,247 | 1,591 | 0,725 | 3,494 |
| **PRO-C8** | **0,022** | **1,091** | **1,012** | **1,176** |

| CA19-9 (OS) | p-value | Hazard ratio | 95,0% CI for hazard ratio | |
| --- | --- | --- | --- | --- |
|  |  |  | Lower | Upper |
| Age | 0,001 | 1,049 | 1,020 | 1,080 |
| Gender (male 0, female 1) | 0,263 | 0,674 | 0,339 | 1,344 |
| Height | 0,451 | 0,994 | 0,978 | 1,010 |
| Weight | 0,789 | 0,996 | 0,968 | 1,025 |
| CEA | 0,645 | 1,000 | 0,999 | 1,001 |
| Metastases (no 0, yes 1) | 0,140 | 1,740 | 0,834 | 3,629 |
| ***CA19-9*** | ***0,078*** | ***1,000*** | ***1,000*** | ***1,000*** |

| C3M (OS) | p-value | Hazard ratio | 95,0% CI for hazard ratio | |
| --- | --- | --- | --- | --- |
|  |  |  | Lower | Upper |
| Age | 0,001 | 1,056 | 1,023 | 1,089 |
| Gender (male 0, female 1) | 0,379 | 0,734 | 0,368 | 1,464 |
| CA19-9 | 0,018 | 1,000 | 1,000 | 1,000 |
| Height | 0,446 | 0,994 | 0,978 | 1,010 |
| Weight | 0,883 | 1,002 | 0,973 | 1,032 |
| CEA | 0,746 | 1,000 | 0,999 | 1,001 |
| Metastases (no 0, yes 1) | 0,257 | 1,531 | 0,733 | 3,200 |
| ***C3M*** | ***0,001*** | ***1,043*** | ***1,016*** | ***1,070*** |

| PRO-C3 (OS) | p-value | Hazard ratio | 95,0% CI for hazard ratio | |
| --- | --- | --- | --- | --- |
|  |  |  | Lower | Upper |
| Age | 0,003 | 1,047 | 1,016 | 1,079 |
| Gender (male 0, female 1) | 0,320 | 0,702 | 0,349 | 1,411 |
| CA19-9 | 0,844 | 1,000 | 1,000 | 1,000 |
| Height | 0,936 | 1,001 | 0,984 | 1,017 |
| Weight | 0,248 | 0,983 | 0,955 | 1,012 |
| CEA | 0,614 | 1,000 | 0,999 | 1,001 |
| Metastases (no 0, yes 1) | 0,040 | 2,285 | 1,038 | 5,027 |
| ***PRO-C3*** | ***0,005*** | ***1,002*** | ***1,001*** | ***1,004*** |

| rec1M (OS) | p-value | Hazard ratio | 95,0% CI for hazard ratio | |
| --- | --- | --- | --- | --- |
|  |  |  | Lower | Upper |
| Age | <0,001 | 1,072 | 1,034 | 1,112 |
| Gender (male 0, female 1) | 0,080 | 0,539 | 0,270 | 1,077 |
| CA19-9 | 0,016 | 1,000 | 1,000 | 1,000 |
| Height | 0,619 | 0,995 | 0,978 | 1,014 |
| Weight | 0,878 | 0,998 | 0,967 | 1,029 |
| CEA | 0,907 | 1,000 | 0,999 | 1,001 |
| Metastases (no 0, yes 1) | 0,190 | 1,648 | 0,780 | 3,478 |
| ***reC1M*** | ***0,034*** | ***1,007*** | ***1,000*** | ***1,013*** |

**
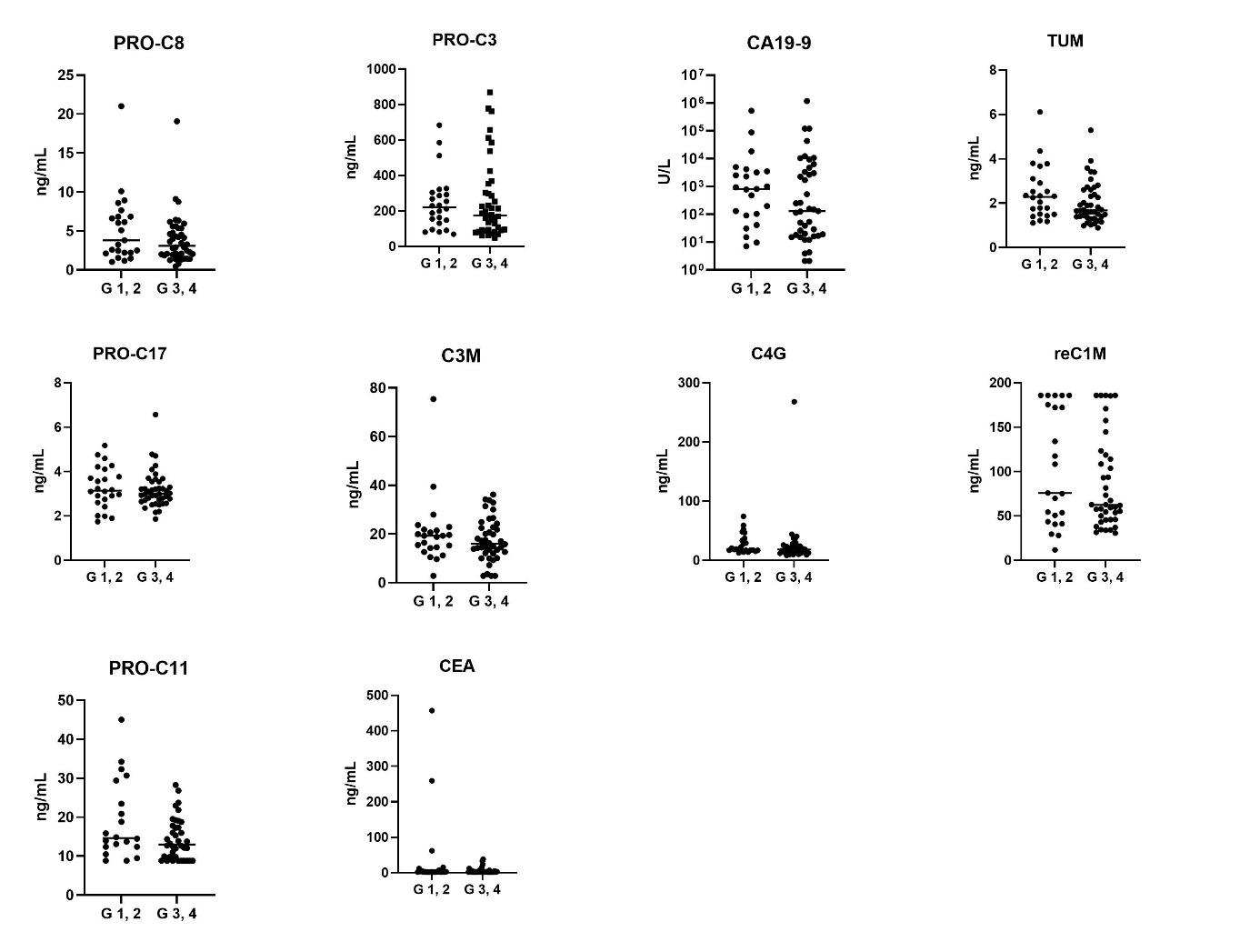
**

SI figure 1. Quantification of the collagen turnover markers in sera of patients with biliary tract cancer (BTC) stratified for tumor histological grading G 1, 2 versus G 3, 4 at baseline (the bars in the graphs represent the median).


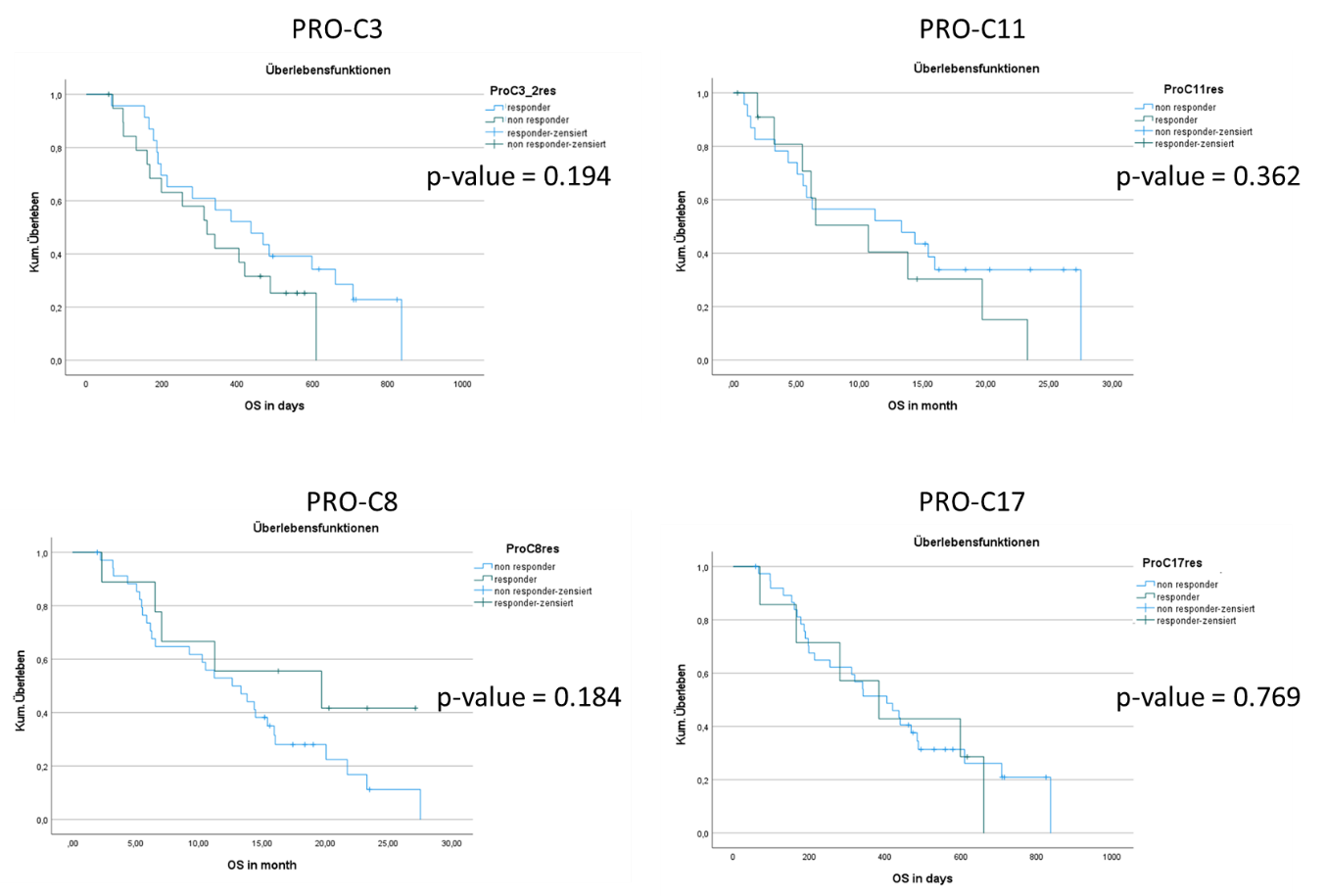


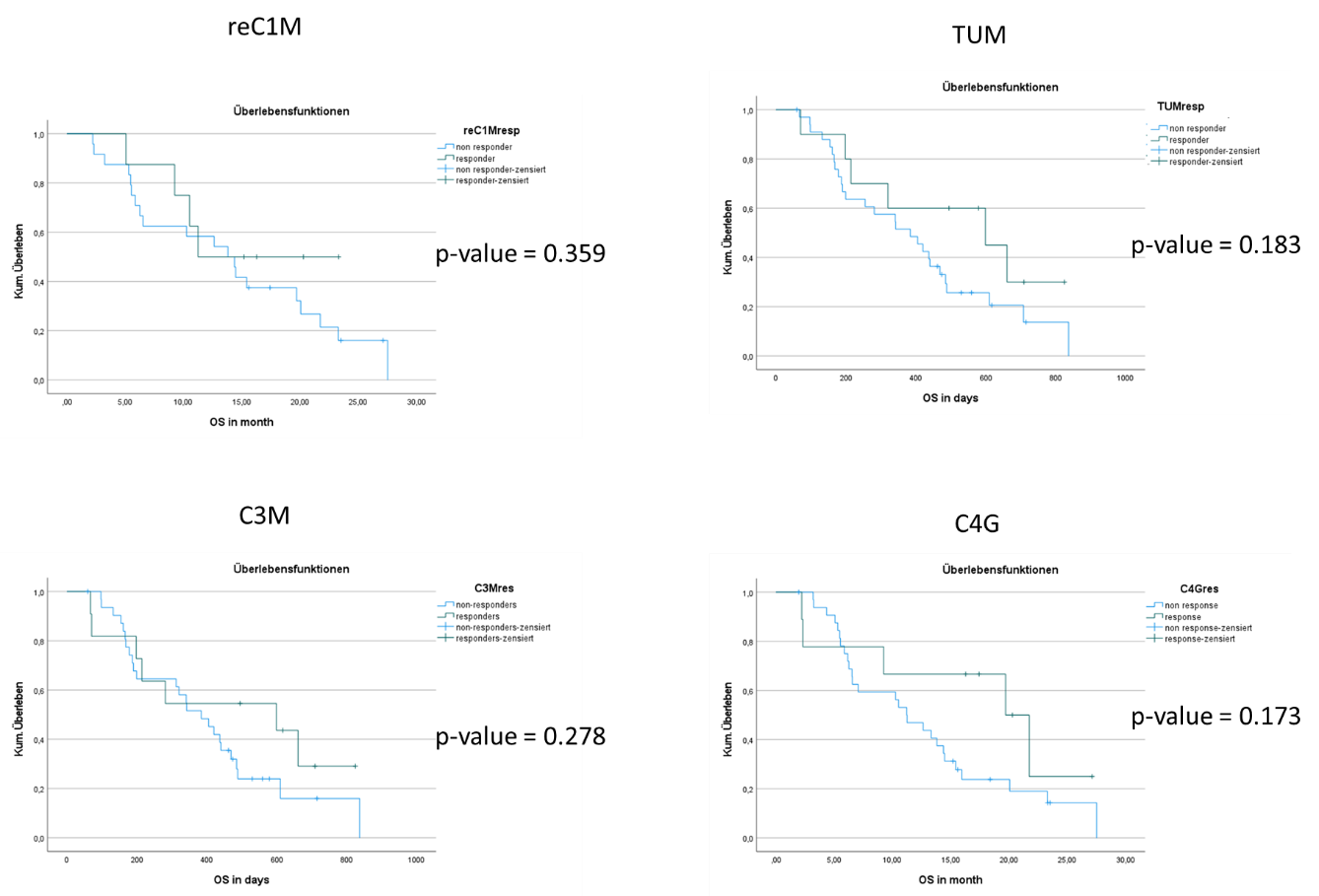


SI figure 2. Kaplan-Meier survival plots of patients with biliary tract cancer (BTC) who responded to chemotherapy with a ≤20 % reduction of biomarker levels after first chemotherapy compared to baseline versus non-responders.
